# Obesity and Lifestyle Habits among Kidney Transplant Recipients

**DOI:** 10.3390/nu14142892

**Published:** 2022-07-14

**Authors:** Maria Grazia Tarsitano, Gabriele Porchetti, Rossana Caldara, Antonio Secchi, Caterina Conte

**Affiliations:** 1 Nutrition Unit, Department of Medical and Surgical Science, University Magna Grecia, 88100 Catanzaro, Italy; mariagrazia.tarsitano@unicz.it; 2 Department of Human Sciences and Promotion of the Quality of Life, San Raffaele Roma Open University, Via di Val Cannuta 247, 00166 Rome, Italy; gabriele.porchetti@libero.it; 3 Division of Immunology, Transplantation and Infectious Diseases, IRCCS San Raffaele Hospital, Via Olgettina 60, 20132 Milan, Italy; caldara.rossana@hsr.it (R.C.); secchi.antonio@hsr.it (A.S.); 4 School of Medicine, Vita-Salute San Raffaele University, Via Olgettina 58, 20132 Milan, Italy; 5 Department of Endocrinology, Nutrition and Metabolic Diseases, IRCCS MultiMedica, Via Milanese 300, Sesto San Giovanni, 20900 Milan, Italy

**Keywords:** obesity, kidney transplant, Mediterranean diet, physical activity, lifestyle

## Abstract

Background: Obesity may negatively impact clinical outcomes in kidney transplant (KT) recipients. Limited information is available on the prevalence of obesity in this population, and on the lifestyle habits associated with obesity. Methods: we conducted an online, anonymous survey to assess of the proportion of KT recipients with obesity, adherence to the Mediterranean diet (i.e., a dietary regimen with proven renal and cardiovascular outcomes) using the MEDI-Lite questionnaire, and level of physical activity using the International Physical Activity Questionnaire (IPAQ) short form among KT recipients. Results: 255 KT recipients participated. Median (25th–75th quartile) age was 56.0 (48.0; 62.0) years, 43.9% female, median BMI 23.9 (21.6; 26.5) kg/m^2^. The proportion of KT recipients with obesity was 9.8% (95% confidence interval, 6.4 to 14.1%). Adequate adherence to the Mediterranean diet (Medi-Lite score >9) was overall low (44.7%; 40.0 vs. 45.2% in those with or without obesity, respectively; *p* = 0.618). In participants with obesity the Medi-Lite score inversely correlated with BMI (R = −0.45; *p* < 0.025). Overall, 30.6% of participants had a low level of physical activity (44.0 vs. 29.1% of those with or without obesity, respectively; *p* = 0.125). The amount of energy expended walking was significantly lower among participants with obesity (462 (0.0; 1436) vs. 1056 (433; 2005) METs/week, *p* = 0.017). Conclusions: the burden of obesity among KT recipients is similar to that of the general population. Adherence to the Mediterranean diet was generally low, and nearly one-third of participants had a low level of physical activity. Building specialized multidisciplinary teams to manage obesity in KT recipients is urgently needed.

## 1. Introduction

Obesity is a complex, chronic disease that impacts nearly all organs and systems, including the urinary system [1]. The prevalence of obesity and chronic kidney disease (CKD) has increased steeply over the past decades [2,3,4], and the coexistence of the two diseases is common [5]. Obesity is an independent risk factor for the development and progression of CKD, and poses significant challenges in the management of CKD patients [6]. Of note, obesity often represents a barrier to kidney transplant (KT), and may impact KT outcomes [7,8,9]. The prevalence of obesity and obesity-associated chronic metabolic alterations is constantly growing among KT recipients, mirroring and even exceeding the prevalence in the general population [7].

Steroid therapy may contribute to post-transplant weight gain [10], but the role of lifestyle factors such as diet and physical activity is also important [11,12], and it is recommended that weight-management programs for KT recipients living with obesity include lifestyle modifications [13]. Several dietary patterns may have favorable effects on health status, the Mediterranean diet being the pattern supported by the strongest evidence with regard to overall mortality, cardiovascular and metabolic diseases [14,15,16]. Furthermore, besides its nutritional value, the Mediterranean diet takes into account conviviality and physical activity [17]. Despite evidence indicating that the Mediterranean diet could slow the progression of CKD and prevent cardiometabolic alterations even in patients with CKD [18], data in the KT population are scarce. Combining a healthy dietary pattern with moderate physical activity, which has independent beneficial effects on cardiometabolic outcomes, may further improve patient and graft outcomes [7,19]. To date, few data are available on lifestyle habits and their association with obesity in the KT population.

Given the impact of obesity on renal function and health status in KT recipients, and the potential benefits associated with a healthy lifestyle (e.g., adherence to the Mediterranean diet and maintaining an adequate level of physical activity), providing information on the burden of obesity and the lifestyle habits of KT recipients is crucial to put the spotlight on an issue that is often overlooked both by healthcare professionals and patients, and to help design targeted strategies aimed at improving patient and graft outcomes. Therefore, the aim of this cross-sectional study was to investigate these aspects in a real-world population of KT recipients.

## 2. Materials and Methods

### 2.1. Study Design

We conducted an anonymous, open online survey among adult (age ≥ 18 years) individuals on renal replacement therapy (either dialysis or KT). The survey was posted on a dedicated web page in Google Forms between 1 January 2022 and 31 March 2022, and was advertised through social media platforms by the main national associations of patients with polycystic kidney disease (PKD) or on renal replacement therapy. We collected self-reported height and weight to compute body mass index (BMI), age, sex, smoking status, region of residence, level of education, working status, cause of CKD, type, year and number of KT, use of steroid therapy, pre-KT dialysis duration and type. Participants were also asked about weight changes after starting dialysis or KT, and whether a physician had provided recommendations about their weight. The total number of questionnaire items was 45 over a single web page. Participants were required to provide an answer to each item in order to submit the questionnaire; therefore, only questionnaires with complete answers were available for analysis. As the two populations have different characteristics (e.g., as compared with KT recipients, patients on dialysis may be subject to more severe dietary restrictions, and might have greater mobility limitations), data were analyzed separately. For the purpose of the present study, only KT recipients were included in the analysis.

### 2.2. Adherence to the Mediterranean Diet

Adherence to the Mediterranean diet was assessed using the MEDI-LITE questionnaire, a validated questionnaire that assesses the consumption of nine food categories (fruit, vegetables, legumes, cereals, fish, meat and meat products, dairy products, alcohol, and olive oil) [20,21]. The questionnaire is validated for use in the Italian population, and yields a score ranging from 0 (minimum) to 18 (maximum). Adherence to the Mediterranean diet was considered inadequate if the score was ≤9, and adequate if >9 [22,23].

### 2.3. Physical Activity

The level of physical activity was assessed using the International Physical Activity Questionnaire (IPAQ) short form, which estimates the level of physical activity (low, moderate, high) based on the subject-reported activities (vigorous/moderate physical activity and walking) relative to the 7 days prior to completion of the questionnaire and is validated for use in the Italian population [24].

### 2.4. Ethical Approval

The study was conducted in accordance with the Declaration of Helsinki, and approved by the Ethics Committee of IRCCS San Raffaele Roma (protocol ODIRT, nr. 21/29). The voluntary questionnaire could be completed only by participants who provided their informed consent to be enrolled in the study. The questionnaire was anonymous; no information that could render the data subject identifiable was collected. The results are reported according to the Checklist for Reporting Results of Internet E-Surveys (CHERRIES) [25]

### 2.5. Statistical Analysis

Descriptive statistics were obtained for all study variables. Normality was assessed with the Shapiro–Wilk test. Continuous variables are expressed as mean ± standard deviation or median (25th–75th percentile), depending on data distribution. Categorical variables were summarized as counts and percentages. Missing data were not imputed. The *t*-test or Mann–Whitney U-test were used for between-group comparisons, depending on variable distribution. The Fisher exact test or the χ^2^ test was used to assess the association between categorical variables and obesity status (BMI ≥ 30 kg/m^2^). The correlation between BMI and the Medi-Lite score was assessed using the Spearman’s rank correlation test. Logistic regression models were used to identify variables associated with obesity, adjusting for age and sex. All variables were screened for violations of the assumptions relevant to each of the statistical analysis performed. Statistical significance was set at *p* < 0.05. Statistical analysis was conducted using IBM SPSS Statistics (IBM SPSS Statistics for Windows, Version 28.0. IBM Corp., Armonk, NY, USA).

## 3. Results

### 3.1. Participant Characteristics

A total of 333 potential participants accessed the informed consent page, of whom eleven (3.3%) did not consent to participate in the study. For the purpose of the present analysis, patients on dialysis (*n* = 67) were excluded. The characteristics of the 255 KT recipients included in the analysis are presented in Table 1.

**Figure 1:**
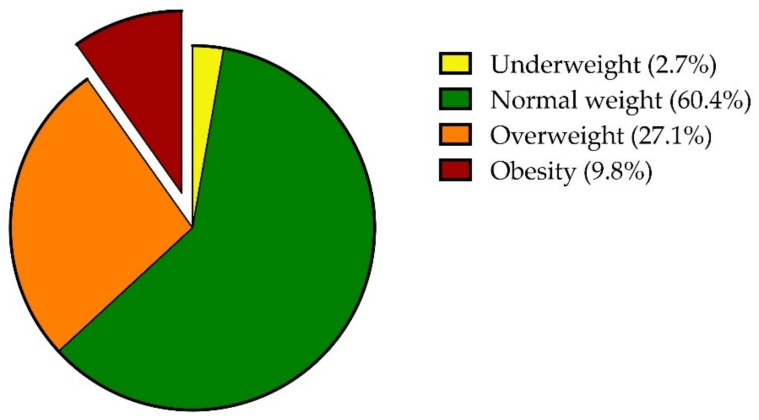
Proportion of participants with underweight, normal weight, overweight and obesity according to BMI values calculated using self-reported height and weight.

The majority of participants were males (56.1%), from Northern Italy, with an intermediate level of education and active workers. PKD was the most common CKD etiology. Nearly all participants received a KT alone, median time since transplant was 6.0 years, and most underwent dialysis prior to KT, for a median of 2.0 years. Hypertension was the most common comorbidity, followed by dyslipidemia, diabetes and vascular disease. Median BMI was 23.9 (21.6; 26.5). The proportion of KT recipients with obesity was 9.8% (95% C.I. 6.4% to 14.1%) (Figure 1), with 19 (7.5%), 3 (1.2%) and 3 (1.2%) participants having obesity classes of I, II and III, respectively.

### 3.2. Comparison between Patients with and without Obesity

A comparison of patients with and without obesity is provided in Table 2. Apart from the median BMI, which markedly differed between groups, there were few significant differences between subjects with and without obesity. The latter were more likely to have undergone dialysis before KT, while participants with obesity were more likely to have metabolic comorbidities such as diabetes and dyslipidemia, and the proportion of participants with hypertension tended to be greater in this group. None of the variables analyzed were significantly associated with obesity at logistic regression.

Participants were surveyed about weight changes following KT. Weight gain was reported by nearly half of the participants (47.8%), whereas 35.3% and 16.9% of participants reported either no change or weight loss after KT. The proportion of participants reporting weight gain after KT was significantly higher among those living with obesity (80.0 vs. 44.3%, *p* = 0.002). All participants living with obesity reported that their physician had addressed their weight and recommended weight loss.

**Figure 2:**
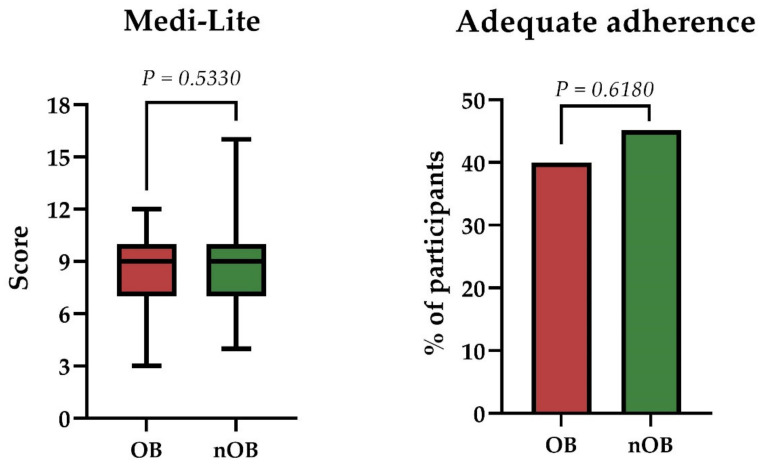
Comparison between patients with (OB) or without (nOB) obesity. Left, Medi-Lite score. Right, proportion of patients with adequate adherence to the Mediterranean diet (defined as a Medi-Lite score >9).

The median Medi-Lite score in the whole cohort was nine (8; 10). There were no significant differences in the Medi-Lite score nor in the proportion of participants with adequate adherence to the Mediterranean diet, which was overall low (44.7%), between participants with or without obesity (Figure 2). Correlation analyses showed a significant inverse correlation between the Medi-Lite score and BMI (R = −0.45; *p* < 0.025) in participants with obesity, but not in the whole cohort nor in participants without obesity.

**Figure 3:**
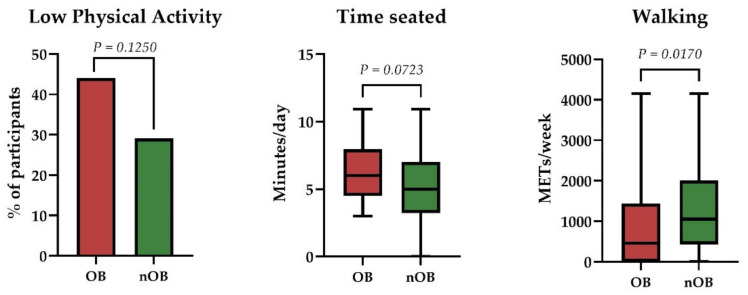
Comparison between patients with (OB) or without (nOB) obesity. Proportion of participants with a low level of physical activity, as assessed by IPAQ-SF (left), time spent seated (center) and amount of energy expended walking.

Analysis of the IPAQ-SF responses revealed that, overall, 30.6% of participants had a low level of physical activity. This proportion was numerically higher among participants with obesity, as compared to those without obesity (Figure 3). Furthermore, participants with obesity spent more time seated, this difference being borderline significant (Figure 3). The level of physical activity, as assessed by metabolic equivalents, was similar between groups except for the amount of energy expended walking, which was significantly lower among participants with obesity (Figure 3).

## 4. Discussion

We assessed the proportion of KT recipients with obesity and important lifestyle habits, including the adherence to Mediterranean diet and the level of physical activity, among KT recipients. We found that approximately 10% of KT recipients who participated in the survey were living with obesity. This figure is in line with recent data from the Italian National Institute of Health (Istituto Superiore di Sanità, ISS, Rome, Italy), which reports that 10.8% of Italians live with obesity [27], but is lower when compared to other cohorts of KT recipients from other countries, where the prevalence of obesity ranged from 20% in Germany [9] to 31% in the United States [28], likely due to different prevalence rates of obesity in the general population. Weight gain after KT was reported by most (80%) participants living with obesity in our cohort. It has long been recognized that weight gain after KT occurs in a significant proportion of patients [29]. Our data are consistent with a recent analysis showing that ~73% of KT recipients gain weight one year after KT, with female sex and pre-transplant body weight being independent predictors of clinically significant weight gain [30]. We were unable to identify factors associated with obesity in our cohort. Longer dialysis duration is a risk factor for sarcopenia (i.e., reduced muscle mass and function) in KT recipients [31], which might explain our finding of longer pre-transplant dialysis in participants without obesity. Previous evidence indicates that other risk factors for weight gain after KT include a younger age, black ethnicity, lower socioeconomic status, diabetes mellitus, acute rejection, steroids, and antidepressants [29]. Post-transplant weight gain is due to increases in both fat and fat free mass [32]. However, KT recipients exhibit persistent relative sarcopenia, as the gain in fat mass outpaces the improvements in muscle mass and strength, which remain low when compared to healthy controls [32]. These observations highlight the need for screening KT recipients not only for obesity, but also for altered body composition, this population being at increased risk of sarcopenic obesity [33].

Most available data indicate that KT recipients with obesity are at risk of worse patient and graft outcomes, with the risk progressively increasing with increasing BMI [13]. The risk may be mediated by comorbid factors, particularly cardiovascular risk factors. Cardiometabolic disturbances such as diabetes, dyslipidemia and hypertension were more common among participants with obesity in our cohort. These alterations are typically associated with obesity, and are also risk factors for reduced kidney graft survival [34]. As KT recipients are at an increased cardiovascular risk [35], it is of the utmost importance to reduce the burden of modifiable risk factors such as obesity and obesity-associated cardiometabolic disturbances. In this light, having a “healthy” lifestyle plays a pivotal role.

We surveyed participants about their adherence to the Mediterranean diet using the validated Medi-Lite questionnaire. The median Medi-Lite score in our cohort was nine, which is three points lower than that found in a sample of nearly 2000 Italian adults by Dinu and colleagues [20], and one point lower than that found by the same researchers in 280 patients with overweight/obesity [22], suggesting a lower adherence among KT recipients as compared with the general population. In fact, adequate adherence to the Mediterranean diet was overall low, with less than half of participants scoring more than nine on the Medi-Lite questionnaire. There was no significant difference between individuals with or without obesity, possibly due to the overall low level of adherence. However, we found a significant inverse correlation between the Medi-Lite score and BMI (R = −0.45; *p* < 0.025) in participants with obesity, which was very similar to that found by Dinu and colleagues in subjects with overweight/obesity (R = −0.41; *p* < 0.0001) [22]. Efforts should be made to improve adherence to the Mediterranean diet among KT recipients. Previous evidence indicates that the Mediterranean diet could help reduce the risk of post-transplantation diabetes and the metabolic syndrome in KT recipients [36,37], although the quality of available data is low due to heterogeneity of interventions, as well as the duration and outcomes of these studies [38]. More recently, a study involving 632 KT recipients followed-up for a median of 5.4 years showed that an increasing adherence to Mediterranean diet was associated with a lower risk of graft failure and kidney function decline, as well as graft loss [39]. Finally, adherence to Mediterranean diet was associated with better nutritional status (greater albumin levels and skeletal muscle mass) in Dalmatian KT recipients [40].

Physical activity, together with healthy eating, is a pillar of the management of obesity and associated cardiometabolic alterations. A recent analysis found that an active lifestyle is associated with slower decline of renal function in KT recipients [41]. We found that nearly one third of participants had a low level of physical activity, this proportion being numerically greater among those with obesity. Furthermore, participants with obesity spent more time seated, and expended less energy walking, suggesting a more sedentary lifestyle in this group. This finding is consistent with data from the Italian National Transplant Center showing that KT recipients with obesity were less likely to be physically active, defined as engaging in physical activity ≥30-min, 5 times/week [41]. Reduced physical activity and a more sedentary lifestyle have been previously reported in KT recipients [42], and have been identified as factors contributing to post-transplant weight gain [11,43]. Adopting a healthy lifestyle is key to achieving and successfully maintaining weight loss. However, it should be recognized that obesity is a complex disease, and several mechanisms counteract weight loss and weight maintenance after weight loss, such as the action of gut hormones that stimulate food intake and the depression of energy expenditure [44]. Of note, the latter has also been described in KT recipients [11]. Additional KT-specific factors may impact the adherence to a healthy lifestyle in KT recipients, such as a fear of movement due to concerns about hurting the graft [45,46]. These considerations highlight the need for specialized multidisciplinary teams including obesity and sports medicine physicians able to provide lifestyle counselling and—when indicated—pharmacological therapy or referral to bariatric surgery.

Some limitations of our study need to be acknowledged. To keep the survey completely anonymous, we did not collect information (e.g., IP address, cookies) that could be used to identify duplicate entries, therefore we cannot exclude that multiple entries from the same individual were included in the analysis. It is possible that the actual proportion of KT recipients with obesity is even higher than that we found, as self-reported height tends to be overestimated and weight to be underestimated when compared with measured values [47]. Furthermore, due to the nature of our study, we were not able to assess the body composition of participants. The proportion of participants from Central and Southern Italy was relatively low, limiting the applicability of our findings. Finally, the primary goal of this study was to assess the proportion of KT recipients with obesity, therefore it is possible that there was inadequate power to detect associations between obesity and lifestyle factors.

## 5. Conclusions

In conclusion, the burden of obesity among KT recipients is similar to that of the general Italian population. Adherence to the Mediterranean diet was generally low, and nearly one-third of participants had a low level of physical activity. Obesity in KT recipients may be associated with a lower adherence to the Mediterranean diet and reduced physical activity. Building specialized multidisciplinary teams to manage obesity in KT recipients is urgently needed.

## Figures and Tables

**Table 1:** Participant characteristics.

Variable	Value
Age, years	56.0 (48.0; 62.0)
Sex (female), *n* (%)	112 (43.9)
BMI, kg/m^2^	23.9 (21.6; 26.5)
Active smoker, *n* (%)	17 (6.7)
Geographical area, *n* (%) - Northern Italy - Central Italy - Southern Italy	181 (71.0) 38 (14.9) 36 (14.1)
Level of education ^1^, *n* (%) - Basic - Intermediate - Advanced	39 (15.3) 130 (51.0) 85 (33.3)
Active worker, *n* (%)	132 (51.8)
CKD etiology, *n* (%) - Hypertension - Diabetes - PKD - Autoimmune disease - Other/unknown	10 (3.9) 41 (16.1) 104 (40.8) 26 (10.2) 74 (29.0)
KT type, *n* (%) - Kidney alone - Kidney-pancreas - Kidney-liver	251 (98.4) 3 (1.2) 1 (0.4)
Transplant vintage, years	6.0 (3.0; 13.0)
Number of KTs, *n* (%) - One - Two	243 (95.3) 12 (4.7)
Steroid therapy, *n* (%)	132 (52.0)
Dialysis prior to KT, *n* (%)	194 (76.1)
Dialysis prior to KT, years	2.0 (1.0; 4.3)
Comorbidities, *n* (%) - Hypertension - Diabetes - Dyslipidemia - Vascular disease ^2^	151 (59.2) 50 (19.6) 67 (26.3) 20 (7.8)

^1^ Aggregate levels of education according to the International Standard Classification of Education [26]. ^2^ Includes cardiovascular and cerebrovascular disease. BMI, body mass index, CKD, chronic kidney disease; KT, kidney transplant; PKD, polycystic kidney disease.

**Table 2:** Comparison between patients with (OB) and without (nOB) obesity.

Variable	OB	nOB	*p*-Value
Age, years	54.0 (45.0; 60.0)	56.5 (48.0; 62.0)	0.476
Sex (female), *n* (%)	14 (56.0)	98 (42.6)	0.200
BMI, kg/m^2^	32.9 (31.6; 35.4)	23.4 (21.4; 24.4)	<0.001
Active smoker, *n* (%)	1 (4.0)	16 (7.0)	0.830
Geographical area, *n* (%) - Northern Italy - Central Italy - Southern Italy	20 (80.0) 4 (16.0) 1 (4.0)	161 (70.0) 34 (14.8) 35 (15.2)	0.217
Level of education ^1^, *n* (%) - Basic - Intermediate - Advanced	5 (20.0) 11 (44.0) 9 (36.0)	34 (14.8) 119 (51.7) 76 (33.0)	0.823
Active worker, *n* (%)	15 (60.0)	117 (50.9)	0.386
CKD etiology, *n* (%) - Hypertension - Diabetes - PKD - Autoimmune disease - Other/unknown	0 (0.0) 6 (24.0) 9 (36.0) 5 (20.0) 5 (20.0)	10 (4.3) 35 (15.2) 95 (41.3) 21 (9.1) 69 (30.0)	0.189
KT type, *n* (%) - Kidney alone - Kidney-pancreas - Kidney-liver	25 (100.0) 0 (0.0) 0 (0.0)	226 (98.3) 3 (1.3) 1 (0.4)	0.660
Transplant vintage, years	6.0 (4.0; 16.5)	6.0 (3.0; 13.0)	0.368
Number of KTs, *n* (%) - One - Two	25 (100.0) 0 (0.0)	218 (94.8) 12 (5.2)	0.614
Steroid therapy, *n* (%)	11 (44.0)	121 (52.8)	0.401
Dialysis prior to KT, *n* (%)	14 (56.0)	180 (78.3)	0.013
Dialysis prior to KT, years	3.5 (1.0; 7.3)	2.0 (1.0; 4.0)	0.407
Comorbidities, *n* (%) - Hypertension - Diabetes - Dyslipidemia - Vascular disease ^2^	19 (76.0) 12 (48.0) 12 (48.0) 3 (12.0)	132 (57.4) 38 (16.5) 55 (23.9) 17 (7.4)	0.072 <0.001 0.009 0.427

^1^ Aggregate levels of education according to the International Standard Classification of Education [26]. ^2^ Includes cardiovascular and cerebrovascular disease. BMI, body mass index, CKD, chronic kidney disease; KT, kidney transplant; PKD, polycystic kidney disease.

## Data Availability

The data presented in this study are available on request from the corresponding author.
